# Disabled or Cyborg? How Bionics Affect Stereotypes Toward People With Physical Disabilities

**DOI:** 10.3389/fpsyg.2018.02251

**Published:** 2018-11-20

**Authors:** Bertolt Meyer, Frank Asbrock

**Affiliations:** Chemnitz University of Technology, Chemnitz, Germany

**Keywords:** stereotypes, physical disabilities, bionics, prosthetics, open data

## Abstract

According to the Stereotype Content Model that construes attributions of warmth and competence as the core dimensions of stereotypes, people with physical disabilities are generally perceived as warm-but-incompetent, i.e., are faced with paternalistic stereotypes. We argue that the increasing proliferation of bionic technologies (e.g., bionic arm and leg prostheses, exo-skeletons, retina implants, etc.) has the potential to change stereotypes toward people with physical disabilities: The portrayal of people who use such devices in the media and popular culture is typically characterized by portraying them as competent - sometimes even more competent than able-bodied individuals. We thus propose that people with physical disabilities who use bionic prostheses are perceived as more competent than people with physical disabilities in general. We also propose that they can be seen as more competent than able-bodied individuals. We further propose that this increase in perceived competence may be associated with a decrease in warmth such that people who use bionic prostheses are perceived as less warm than people with physical disabilities in general and as able-bodied people. Based on labeling theory, we also propose that using the label “cyborg” for people who use bionic prostheses exacerbates these effects and that they are driven by the technicality of the bionic devices. The first of two online studies (*n* = 314) revealed mixed support for the hypotheses: People with physical disabilities who use bionic prostheses are seen as more competent than people with physical disabilities in general, but not as more competent than able-bodied individuals. They are perceived as even warmer than able-bodied individuals. On the contrary, cyborgs were perceived as competent-but cold, i.e., as threatening. With the second study (*n* = 87), we tested whether the perceived technicality of bionic technology drives some of the observed effects. Technicality only had marginal effects on competence perceptions and no effects on perceptions of warmth. We discuss potential implications and highlight that despite being somewhat mixed, these findings show that technology can affect stereotypes and interpersonal perceptions.

## 1. Introduction

Almost 16% of the global population have a disability (World Health Organization, 2011). People with physical disabilities face generally negative stereotypes toward them (Crocker and Major, 1989); society tends to view physical disability as an inferior state and as a personal misfortune (Thomson, 2017). Therefore, the desire to meet cultural standards for worth of people with physical disabilities is under constant threat (Silverman and Cohen, 2014).

According to the Stereotype Content Model (SCM, e.g., Fiske et al., 2002), which we explain below in more detail, stereotypes about social groups convey information about the groups' intentions (the *warmth* dimension ranging from cold [bad intentions] to warm [good intentions]) and their ability to put their intentions into actions (the *competence* dimension, ranging from incompetent to competent). Prior research (e.g., Cuddy et al., 2009; Asbrock, 2010) shows that people with physical disabilities are typically perceived as having good intentions but as lacking the abilities to put them into action (i.e., as warm-but-incompetent). Perceiving people with physical disabilities in this way typically evokes feelings of sympathy and pity toward them.

The extension of the Stereotype Content Model, the BIAS Map (Behaviors from Intergroup Affect and Stereotypes; Cuddy et al., 2007), postulates that attributions of warmth and competence influence behavior toward these groups. Specifically, it predicts that the warm-but-incompetent stereotype toward people with disabilities leads to active facilitation (helping) and passive harm (ignoring, excluding). Both behaviors signal to the person on the receiving end that they are viewed as incompetent; the chronic experience of this stereotype negatively affects the motivation, performance, and well-being of people with physical disabilities (Silverman and Cohen, 2014).

At the same time, technology for assisting people with physical disabilities has advanced dramatically over the past years. New developments at the intersection of computer science, engineering, robotics, and medicine include exo-skeletons for people with paraplegia, powered and computer-controlled leg prostheses, fully articulate bionic hands, and cochlear implants for people who are deaf. These developments receive widespread media coverage and public attention; a TED-Talk on bionic prostheses (Herr, 2014) currently has more than six million views.

We argue that the increasing proliferation of these bionic technologies has the potential to change stereotypes toward people with physical disabilities: The portrayal of people with disabilities who use such devices in the media and popular culture is typically not characterized by pity. Instead, super villains sporting bionic prostheses (e.g., the assassin “Gazelle” in the 2014 film “Kingsman: The Secret Service”, who can extend swords from her carbon leg prostheses) and record-setting Paralympic athletes (e.g., the long jumper Markus Rehm who won the German Championships against able-bodied athletes in 2014 with his carbon leg prosthesis) are portrayed as anything-but incompetent. Modern high-tech prostheses and exo-skeletons are designed to highlight their technological nature and features. Therefore, we propose that such bionic devices have the potential to change how people with physical disabilities who rely on them are perceived: The high-tech devices signal competence, which substitutes the notion of incompetence that is typically conveyed by physical disabilities. On the basis of the Stereotype Content Model, we thus argue that people with physical disabilities who use bionic prostheses are perceived as more competent than people with physical disabilities in general.

If technology can change attributions of competence toward people with physical disabilities, it is also likely to affect attributions of warmth: Prior research shows that when an ambivalently stereotyped group advances on one dimension (e.g., competence), it is likely to be devalued on the other (e.g., warmth, Cuddy et al., 2004). This shift in attributions can be reflected in a shift in labels, because language can influence perceptions of social groups (Sapir, 1921). For example, a recent study (Kotzur et al., 2017) showed that the evaluation of refugees in terms of warmth and competence depends on the label describing them (e.g., refugees, asylum seekers, war refugees, economic refugees). In line with this reasoning, we propose that the label that one uses for describing individuals with physical disabilities who use bionic technologies affects attributions of warmth and competence toward such individuals. Specifically, we propose that labeling these individuals as “cyborgs” can paint them as competent-but-cold. We further propose that perceptions of warmth and competence is related to the perceived technicality of the assistive devices.

In sum, we believe that our study can make a valuable contribution to research on stereotypes and stigma by showing if and how technology has the potential to affect them.

## 2. Disabled, able-bodied, therapy, and enhancement: working definitions

For discussing the impact of technology on the perceptions of people with disabilities, it is necessary to define the terms disability and therapy and to distinguish between therapeutic and assistive technology on the one hand and enhancement on the other. Disability implies a deviation from what is considered “normal” (Davis, 1995). In the context of disability and illness, “normal” is typically associated with a frequentist meaning of the word, in the sense of “common” (Davis, 1995). In other words, the range of most frequent occurrences of an ability (e.g., average eyesight) is set as the norm. As a consequence, someone is considered ill or disabled if their capacity falls below a certain threshold. As an example, individuals are considered “legally blind” if their visual acuity is below 20/200—i.e., if a person can see details of an object that is 20 feet away that a person with normal eyesight can see from 200 feet away (American Foundation for the Blind, 2017). Therefore, in this institutionalized or medical/disablelist view of disability, disability is an issue that lies within the person: The person's ability is so far below the population mean that it falls below the threshold of what is considered “normal”.

The difference between enhancement and therapy rests on their respective goals and the conditions they attempt to modify (Allhoff et al., 2010; Menuz et al., 2013). Therapy attempts to alleviate a burden caused by a disability or illness (Missa and Perbal, 2009). Enhancement has been defined as “the result of the application of NBICs [nanotechnology, biotechnology, information technology and cognitive science] to individuals so as to improve their body, mind or any ability beyond the species-typical level or statistically-normal range of functioning of a human being” (Menuz et al., 2013, p. 162). In short, therapy is about enhancing the disabled state, while enhancement is about enhancing the able-bodied “normal” state: “Conventionally, therapeutic interventions are understood to restore or bring an individual's morphology and capacities within the normal range, while enhancements imply going beyond what is normal” (Karpin and Mykitiuk, 2008, p. 414). Therefore, these distinctions suggest that disability refers to a state or capacity below the statistically normal range, while enhancement refers to a state above this range.

This normative or medical model of disability can be contrasted with a social model of disability (Shakespeare, 2006), that does not attribute normalcy to the frequency of a certain capacity, but construes all occurrences of a capacity, no matter how rare, as normal. While the medical model of disability sees the problem of disability in the under-capacity of the body, the social model of disability conceptualizes disability as the mismatch between the abilities of the individual (the impairment of the individual) and environmental and/or societal expectations toward the individual. For example, being unable to walk is a disability from the perspective of the normative model, while the social model of disability assumes that the disability of this state arises from the ubiquitousness of stairs. If there were ramps everywhere, sitting in a wheelchair would not constitute a problem. Only the ubiquitousness of stairs and their implied expectation that one is able to walk constitutes the mismatch between expectations and ability in this case, which results in disability. Therefore, interventions seeking to overcome disability that are based on the medical/normative model of disability can only target the body for overcoming disability, while interventions that are based on the social model of disability can target both the body and the environment. Current conceptualizations of disability are however typically based on the psychosocial model of disability (World Health Organization, 2011), which construes disability as difficulties in the areas of body function, activities, and involvement in any area of life. As such, “disability arises from the interaction of health conditions with contextual factors—environmental and personal factors” (World Health Organization, 2011, p. 5).

## 3. Stereotypes toward people with disabilities: the stereotype content model

Stereotypes are socially shared beliefs about members of social groups that disregard individuality (Jonas et al., 2002). Stereotypes say, for example, that Italians make good pasta and that senior citizens are hard of hearing. According to the Stereotype Content Model (Fiske et al., 2002), stereotypes convey information on two key dimensions: warmth (the intentions of members of a social group—from bad [cold] to good [warm]) and competence (how well members of a social group can put their intentions into action—from bad [incompetent] to good [competent]). Put simply, four kinds of stereotyped groups arise: First, the competent and warm ones. These are generally the society's reference or default groups, for example, citizens, the middle class, and the cultural majority (e.g., white heterosexual able-bodied men). These groups receive admiration and pride. The opposite stereotype construes social groups as cold and incompetent, describing the “lowest of the low” (Fiske, 2018, p. 68). Examples include the homeless and drug addicts, who evoke disgust and contempt. In addition to those univalent stereotypes, the SCM depicts two additional stereotypes as ambivalent, because they construe social groups as high on one dimension but low on the other. Groups perceived as warm and incompetent (examples include the elderly and people with physical disabilities), evoke sympathy and pity, whereas competent but cold groups elicit envy and jealousy (in almost all cultures, rich people and bankers are classified here).

Approximately 20 years of research confirm this two-dimensional structure of the contextual meaning of stereotypes (Fiske, 2018). The SCM is universal across nearly 50 sampled countries (Fiske, 2018), but certain culture-specific differences exist. For example, a study from Germany on the SCM (Asbrock, 2010) replicated the finding that housewives tend to be regarded as warm and incompetent, that feminists tend to be seen as cold and competent, and that people with a physical disability are viewed like the elderly, as being warm and incompetent. However, it showed that, differing from the cold-but-competent stereotype in US samples, Jews were perceived as warm and competent. Moreover, certain societal aspects affect the relation between warmth and competence. Durante et al. (2012) showed that countries with higher income inequality are characterized by more ambivalent stereotypes than more equal countries. Another cross-cultural study showed that peaceful countries and those with extreme conflict express less ambivalent stereotypes, while a mediate level of conflict (in countries like the US) is associated with more ambivalent stereotypes (Durante et al., 2017).

As mentioned above, changes in the perception of one dimension can be compensated by a change on the other dimension. In an experimental study, participants rated working women with and without children on warmth and competence. Being a working mother changed the stereotype of the working woman from cold and competent to warm and incompetent and resulted in discriminatory intentions toward the working mother regarding job promotion (Cuddy et al., 2004). This study gives an example of sub typing within the stereotype content model, that is, various subgroups are perceived that do not match the generalizing stereotyped. This leads to sub types of the category with more specific stereotypes—the warm and incompetent housewife, the cold and competent career woman. Various studies describe such sub types of more general groups, for example for males and females (Eckes, 2002), immigrants (Lee and Fiske, 2006), people with mental illnesses (Sadler et al., 2012), and Native Americans (Burkley et al., 2017).

Regarding the perception of people with disabilities, research indicates that incompetence is one of the most common descriptors (Coleman et al., 2015). While most studies in the SCM framework focused on explicit stereotypes, Rohmer and Louvet (2012) showed that the warm-and-competent stereotype of people with physical disabilities shows on the explicit, but not on the implicit level, which provide a more negative stereotype. The authors interpret this as an indication of social desirability in explicit stereotypes of people with physical disabilities. In a study on the perception of children with disabilities, the authors showed that active children with disabilities were perceived as more competent than inactive children, indicating that activity can affect the stereotype of competence (Barg et al., 2010). There was no effect of activity on perceived warmth.

## 4. Implications of bionic technology for stereotypes about physical disabilities

Modern technology promises to overcome physical disabilities by providing those capabilities that a person with a disability lacks. This view is mirrored in recent statements from evangelists of bionic prostheses. Hugh Herr, an MIT professor who is a bilateral below-knee amputee and who designed an advanced bionic foot and ankle prosthesis famously said: “I don't see disability at all, I see bad technology” (CNN, 2013). As mentioned above, a slew of technological advancements have the potential to overcome the physical incompetencies that are associated with physical disabilities. Such advances include developments in the area of bionic hand prostheses such as the fully articulate TouchBionics Ⓡi-limb, and in the area of legs and feet, such as the Össur ⓇPower Knee, the world's first commercially available electrically powered and computer-controlled knee joint for leg prostheses. Other recent developments include bionic exo-skeletons such as the Ekso Bionics Ekso GT Ⓡ, the reWalk Ⓡsystem, or the REX Ⓡsystem, which promise the ability to walk again for individuals with spinal injuries. There is even a retinal implant system, the SecondSight ⓇArgus 2, that projects a video image (still in very poor resolution) into the visual cortex of individuals with a certain type of acquired blindness.

Especially when it comes to modern prostheses and exo-skeletons, current devices emanate the coolness of high-tech gadgets and bear very little resemblance with the orthopedic appliances from only one or two decades ago. For example, until 2008, the only commercially available electric hand prosthesis was a hand in a fixed and unnatural-looking pinch-grip position that was driven by a single motor. This hand could only grasp between thumb and index finger and had to be worn with a flesh-covered plastic glove that easily stained and made it look somewhat like the hand of a display dummy. These so-called cosmeses are intended to disguise limb loss (Hall and Orzada, 2013). Given that individuals with disabilities often feel shame in relation to their bodily differences (Hall and Orzada, 2013), we propose that, through the futile attempt to conform to a body image of normality, such devices convey this sense of shame, because wearers of cosmeses signal that they are trying to hide their disability—sometimes without doing a very good job at it.

Such devices stand in stark contrast to the current top-of-the-line devices with fully dexterous fingers that are driven by six motors grasp around objects in a natural way. Current bionic hand prostheses, which became commercially available in 2008, are complex pieces of industrial engineering. Some of these hands even come with a mobile app for changing settings. Their manufacturers proudly display their company logos on these devices, which typically come in black or white, and which are worn either without a glove at all or with a transparent one that exposes the technology of the artificial hand to the public eye. These devices do not try to hide anything, but explicitly communicate to their surroundings what they are: beautifully engineered complex technology. This development is not limited to hand prostheses. Modern bionic legs and feet are typically worn without cosmetic coverings that try to pass them off as “real” limbs. Some companies even offer fashionable exchangeable covers for arm and leg prostheses that come in a variety of collections and designs–but not in skin colors. If anything, these covers make prostheses even more visible instead of trying to disguise them. In this way, the prosthesis can become a fashionable accessory.

These modern high-tech prostheses that are designed to highlight their technological nature usually do not evoke a reaction of pity, which is not what the SCM predicts for people with physical disabilities—we propose that the technology aspect of these devices signals competence. Therefore, we propose that such modern bionic prostheses have the potential to change how the people with physical disabilities who wear and use them are perceived: The high-tech prosthesis signals competence, which substitutes the notion of incompetence that is typically conveyed by physical disabilities. We thus hypothesize:

Hypothesis 1a: People with physical disabilities who wear bionic prostheses are perceived as more competent than people with physical disabilities in general

If an ambivalently stereotyped group rises on one of the two dimensions of the SCT as we propose with the previous hypothesis, it is likely to pay for this rise by a decline on the other dimension: A we describe above, female professionals—who are perceived as competent but cold—gain in warmth when they become mothers, but also decrease in competence when they do (Cuddy et al., 2004). Stereotypes facilitate self-affirmation (van Dijk et al., 2017) through negative stereotyping of other groups (e.g., Sinclair and Kunda, 2000). Therefore, if people with physical disabilities gain competence through bionic technology, others are more likely to devalue them on the warmth dimension. We thus propose:

Hypothesis 1b: People with physical disabilities who wear bionic prostheses are perceived as colder than people with physical disabilities in general

Recent media portrayals of advances in the area of bionic prostheses have sometimes conveyed the impression that such devices can constitute an enhancement rather than therapy. Examples of such portrayals of people with physical disabilities can be found in recent narratives surrounding Paralympic athletes. The trailer for the British TV coverage of the 2016 Paralympic Games refers to the Paralympic athletes as “Superhumans”, which implies capacities and competencies beyond the normal state toward a state of overcapacity. In the context of enhancement, this high level of implied competence can come at the cost of being perceived as cold. Thus, portraying people with disabilities who wear bionic prostheses as having capacities beyond what is considered normal comes with the risk of putting people with disabilities who wear prostheses in the competent-but-cold quadrant of the SCM. We thus propose:

Hypothesis 2: People with physical disabilities who wear bionic prostheses are perceived as more competent (2a) and as colder (2b) than able-bodied people

What would the SCM predict for individuals who have undergone enhancement, for cyborgs? Per definition, enhancement implies the alteration of a capacity or ability beyond what is considered normal, beyond frequent, toward a state of overcapacity. Therefore, enhanced individuals are *per se* competent, because the increase of competence beyond the societal standard or norm is at the heart of the definition of enhancement. Given that the establishment and maintenance of a feeling of self-worthiness through comparison with other groups is a fundamental human motive (Tajfel and Turner, 1986; Turner et al., 1987), the only way to distinguish the own un-enhanced “normal” ingroup from the out-group of individuals with enhancements is by means of envious stereotypes. Therefore, people with enhancements are likely to be (stereotypically) perceived as competent-but-cold. Being associated with high levels of competence through enhancement will therefore likely come at the price of being perceived as a threat. We thus propose:

Hypothesis 3: Cyborgs are perceived as more competent (3a) and as colder (3b) than people with physical disabilities in general

Hypothesis 4: Cyborgs are perceived as more competent (4a) and as colder (4b) than able-bodied people

Given that we propose that the technicality of bionic prostheses evokes an attribution of competence (which is likely to decrease attributions of warmth), we deem it necessary to test whether such attributions indeed drive attributions of warmth and competence. We thus propose:

Hypothesis 5: Attributed competence of people with prostheses increases with the level of technicality of their prostheses

Hypothesis 6: Attributed warmth of people with prostheses decreases with the level of technicality of their prostheses

We pre-registered all hypotheses, study designs, and a-priori sample size calculations for the following Studies 1 and 2 on OSF prior to data collection, see https://osf.io/u2srb.

## 5. Study 1

The first study was an online experiment aimed at testing Hypotheses 1 through 4.

### 5.1. Methods

Given that Hypotheses 1 through 4 revolve around the attribution of warmth and competence to certain social groups, we conducted this study similarly to other studies that are based on the SCM and that measure warmth and competence attributions to certain social groups (e.g., Fiske et al., 2002; Asbrock, 2010). The social groups required for testing our hypotheses are able-bodied people, people with physical disabilities who use bionic prostheses, people with physical disabilities, and cyborgs. For comparisons with other studies, we also included four prototypical social categories that are typically included in studies based on the SCM because they represent the four quadrants of the model: Homeless people (cold-incompetent), old people (warm-incompetent), medical doctors (warm-competent), and rich people (cold-competent). For explorative reasons, we also included able-bodied people who use technology to enhance themselves as a further category. In sum, Study 1 thus aimed at eliciting attributions of warmth and competence toward the aforementioned ten social groups.

#### 5.1.1. Participants

We conducted an a-priori power analysis with G^*^Power (Faul et al., 2006) for a within-design with ten measures for each participant. We assumed a medium effect size of *f* = 0.25, an average correlation of *r* = 0.50 among measurements, and set the power to at least 0.95. This resulted in a total sample size of 310 participants.

We recruited adult (i.e., age 18–70) English-speaking participants from Europe and the USA via the online platform Prolific Academic (Palan and Schitter, 2017) and paid them 1 GBP for completing the online questionnaire. We set the number of required participants to 310 valid participants. In other words, the study ran until 310 participants completed the questionnaire and also passed the instructional manipulation check (see below).

Due to a delay in the processing of the data base, the system only capped participation after recording 314 valid response, which we kept. These included 189 women and 118 men; 7 participants did not disclose their gender or selected another category. Average age was 37.90 years, *SD* = 12.44, and 45 participants reported that they had some form of recognized disability. The most frequent level of education was a Bachelor's degree, *n* = 106, followed by high school graduate, diploma, or equivalent, *n* = 48, and some college credit with no degree, *n* = 41. Regarding employment, 156 participants were employed for wages, followed by the self-employed, *n* = 45, students, *n* = 29, and homemakers, *n* = 29. The ethics committee of our university reviewed the study prior to data collection and participants gave written informed consent.

#### 5.1.2. Material and procedure

We used an online survey to measure participants' attributions of warmth and competence toward the ten social groups old people, physicians (medical doctors), rich people, homeless people, people with physical disabilities, people with mental disabilities, people with physical disabilities who wear bionic prostheses, cyborgs, able-bodied people who chose to implant technology (e.g., computer chips) into their bodies to enhance their capabilities, and able-bodied people. We clarified the term “bionic prostheses” by stating that bionic prostheses mimic the original function very closely. We also defined the term cyborg as “a human being with both organic and biomechatronic body parts. The term cyborg applies to an organism that has restored function or enhanced abilities due to the integration of some artificial component or technology that relies on some sort of feedback.” The groups were presented in random order.

For each group, we measured stereotypical attributions of warmth and competence in the same way as in many other studies building on the SCM (e.g., Fiske et al., 2002; Asbrock, 2010). Specifically, for each group, we asked “What do people living in your country think about [social group] in general? To be clear, we are not asking about what you personally think about [social group]. We want to know what you think what people in general think about them. In general, [social group] are perceived as …”, followed by the three adjectives likable, warm, and good-natured, which we averaged into one measure of warmth, and by the three adjectives competent, competitive, and independent, which we averaged into one measure of competence. We presented each adjective with five response options labeled not at all (1), very little (2), somewhat (3), to a great extent (4), and completely (5). Following recent recommendations (McNeish, 2017), we quantified the scales' internal consistency with the Omega Total coefficient, which reached 0.93 for warmth and 0.87 for competence, justifying averaging into the respective scales.

To screen out random clicking and to ensure a thorough reading and understanding of each item, we used an instructional manipulation check (IMC, Oppenheimer et al., 2009). The item read “This is to screen out random clicking. Please ignore the rest of this question and answer “not at all” (1) to all six answer options to this question. What do people living in your country think about people with pets in general?”, followed by the six items measuring warmth and competence. All 87 participants who did not click “not at all” (1) on all of these were deemed invalid and were discarded until we reached the sufficient number of valid responses. The IMC item was presented among the random order of other items measuring warmth and competence. We warned participants about the IMC at the top of the page containing the group items. The warning read: “Please read the instructions and questions on this page carefully. This page contains a check to determine thorough reading. Failing this check waives your prolific reward.” The survey concluded with a page asking for participants' demographic data. The data, analyses, and all study materials are available from the OSF repository at https://osf.io/u2srb.

#### 5.1.3. Data analysis

We obtained ten measurements of warmth and competence (for each of the ten social groups) from each participant, totaling 3,140 measurements. Measurements are thus nested in participants and, consequently, likely exhibit non-independence. Indeed, there was significant between-participant variability for warmth, ICC(1) = 0.16, *F*_(312, 2765)_ = 2.817, *p* < 0.001, ICC(2) = 0.64, and for competence, ICC(1) = 0.02, *F*_(312, 2769)_ = 1.173, *p* = 0.025, ICC(2) = 0.15. In other words, participants differed significantly with regard to the levels of warmth and competence that they generally attributed, which is why we employ mixed models (aka Random Coefficient Modeling or Multilevel Modeling) to account for the nested structure of the data (e.g., Bliese, 2009; Galecki and Burzykowski, 2013).

We used R (Version 3.5.1; R Core Team, 2017) and the R-packages *emmeans* (Version 1.2.2; Lenth, 2017), *gplots* (Version 3.0.1; Warnes et al., 2016), *lme4* (Version 1.1.17; Bates et al., 2015), *MASS* (Version 7.3.50; Venables and Ripley, 2002), *Matrix* (Version 1.2.14; Bates and Maechler, 2017), *multcomp* (Version 1.4.8; Hothorn et al., 2008), *multilevel* (Version 2.6; Bliese, 2016), *MuMIn* (Version 1.40.4; BartoĹ, 2018), *mvtnorm* (Version 1.0.8; Genz and Bretz, 2009), *nlme* (Version 3.1.137; Pinheiro et al., 2017), *papaja* (Version 0.1.0.9842; Aust and Barth, 2018), *plotrix* (Version 3.7.2; Lemon, 2006), *survival* (Version 2.42.3; Therneau and Grambsch, 2000), *TH.data* (Version 1.0.9; Hothorn, 2017), and *userfriendlyscience* (Version 0.7.1; Peters, 2017) for all our analyses.

### 5.2. Results

**Figure 1:**
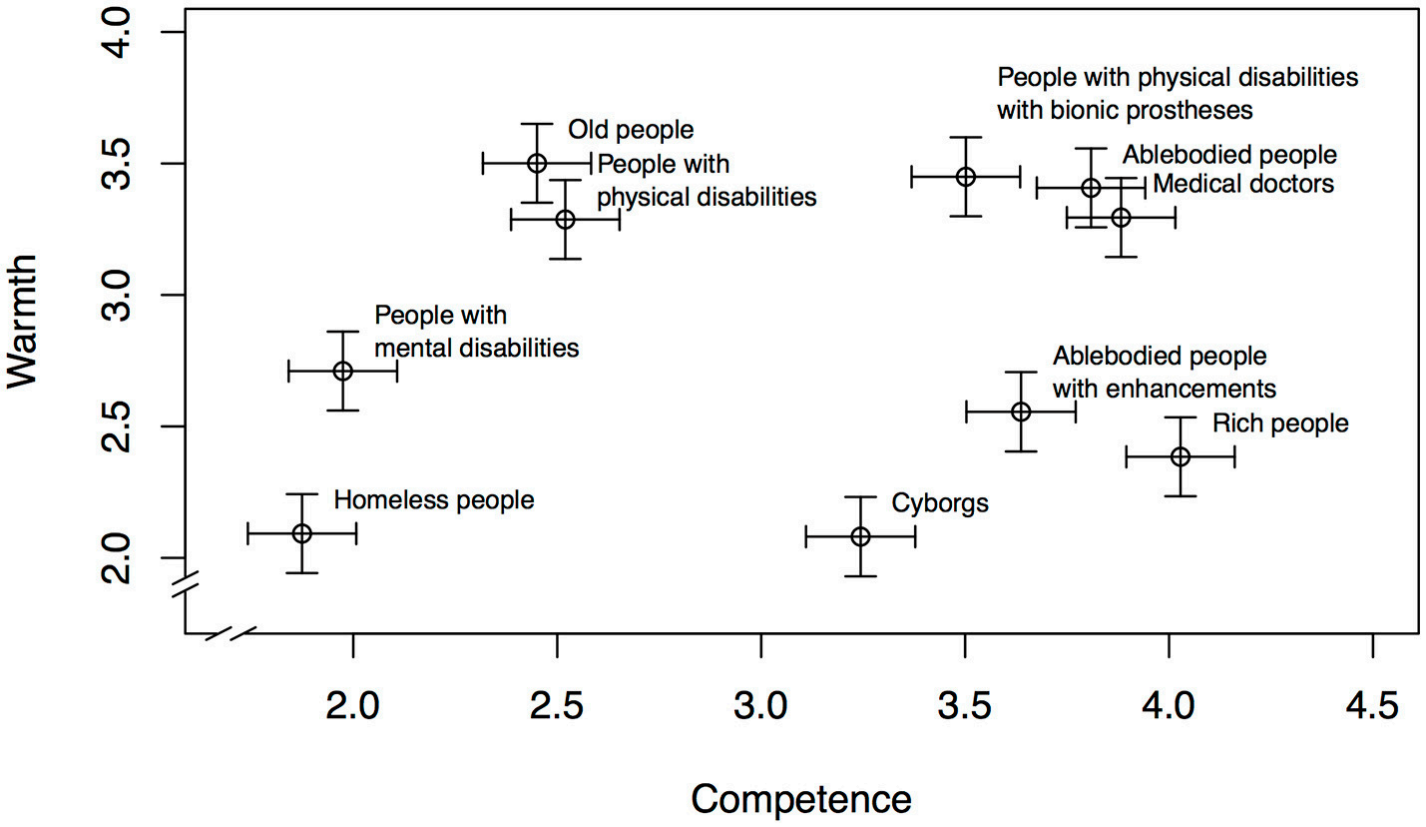
Estimated marginal means of warmth and competence based on the respective mixed models controlling for participants' age, gender, and disability status. Values are based on 3140 observations from 314 participants. Error bars represent 95% confidence intervals.

We fitted two mixed models with random intercepts; one regressed warmth on social group and one regressed competence on social group. In both models, we controlled for participants' gender, age, and disability. The model using warmth as dependent variable revealed that participants' age was negatively associated with ratings of warmth, *b* = −0.01, *SE* = 0.00, *t* = −3.23. Gender and disability were not related to attributions to warmth. The other model that regressed competence on the predictors revealed no significant influences of participants' gender, age, and disability on competence attributions. We estimated both models' marginal means for all ten groups and plotted them into one figure (see Figure 1).

We tested Hypotheses 1-4 with according planned contrasts with Shaffer control for alpha inflation. In support of Hypothesis 1a, participants perceived people with physical disabilities with bionic prostheses as more competent than people with physical disabilities, ΔM = 0.98, *t* = 16.85, *p* < 0.001. Contrary to Hypothesis 2a, participants did not perceive people with physical disabilities with bionic prostheses as more competent than ablebodied people, but the other way around, ΔM = −0.31, *t* = −5.27, *p* < 0.001. In support of Hypothesis 3a, participants perceived cyborgs as more competent than people with physical disabilities, ΔM = 0.72, *t* = 12.30, *p* < 0.001. Rejecting Hypothesis 4a, participants did not perceive cyborgs as more competent than ablebodied people, but the other way around, ΔM = −0.56, *t* = −9.61, *p* < 0.001.

With regard to warmth, in rejection of Hypothesis 1b, participants did not perceive people with physical disabilities with bionic prostheses as colder than people with physical disabilities, but the other way around, ΔM = −0.16, *t* = −3.01, *p* = 0.005. Contrary to Hypothesis 2b, participants also did not perceive people with physical disabilities with bionic prostheses as colder than ablebodied people, ΔM = −0.04, *t* = −0.78, *p* = 0.433. In support of Hypothesis 3b, participants perceived cyborgs as colder than people with physical disabilities, ΔM = 1.21, *t* = 22.14, *p* < 0.001. Supporting Hypothesis 4b, participants also perceived cyborgs as colder than ablebodied people, ΔM = 1.33, *t* = 24.37, *p* < 0.001.

### 5.3. Discussion of study 1

The results partially support our hypotheses: People with physical disabilities who wear bionic prostheses and Cyborgs are perceived as more competent than people with physical disabilities in general. Contrary to what we had expected, these two groups are however not perceived as more competent than able-bodied individuals. Contrary to the hypotheses, participants perceived people with physical disabilities as warm as able-bodied people. Finally, as we had hypothesized, so-called cyborgs are perceived as much colder than able-bodied individuals–in fact, participants perceived cyborgs as cold as homeless people.

Apparently, the increase in competence for people with physical disabilities who wear bionic prostheses in comparison with people with disabilities in general does not come at the cost of decreased warmth. The fact that—contrary to our hypotheses—participants saw people with physical disabilities who wear bionic prostheses as slightly less competent than able-bodied people is one possible explanation: Perceived competition between social groups typically facilitates devaluation of other groups in terms of warmth (e.g., Kervyn et al., 2015). Presumably, people with physical disabilities who wear bionic prostheses do not threat the competence of the able-bodied (yet). In addition, Barg et al. (2010) did not find a negative effect of activity of children with disabilities on perceptions of warmth either.

We were surprised to find that cyborgs and able-bodied people who chose to enhance themselves were both perceived as less competent than able-bodied individuals. This finding is difficult to explain. The term cyborg probably leaves a lot of room for personal interpretations that may involve a certain clumsiness. Given that cyborgs are portrayed in a negative, ugly, and often scary way (e.g., in the “Star Trek” series and films), the term may even come across as somewhat insulting. However, the label “able-bodied people who chose to enhance themselves” clearly implies potential abilities beyond the able-bodied norm. We can only speculate that participants devalue people from this category for enhancing themselves beyond able-bodied, and that this devaluation not only affects perceptions of warmth, but also of competence. We revisit this issue in the general discussion.

In sum, the results of Study 1 show that bionic prostheses do not only have a functional benefit, but also a potential psychological one: People with physical disabilities who use such devices are seen as warm as able-bodied people and almost as competent. However, the label that people use for such individuals is important: If they are called cyborgs instead of people with disabilities who use bionic prostheses, others perceive them as much colder than able-bodied individuals (and hence as a potential threat).

To shed some light on these issues, we investigate whether the capabilities and technological appearance of bionic technology affects the perception of their users in the following.

## 6. Study 2

Hypotheses 5 and 6 predict that the levels of warmth and competence that we partly observed for people with disabilities who wear bionic prostheses and for cyborgs are caused by the technicality of the prosthetic devices. After pre-registering the study on OSF, we tested the hypotheses with an online experiment as we describe in the following.

### 6.1. Methods

We manipulated the technicality of prosthetic devices with a 3 (little technicality/medium technicality/high technicality) × 3 (type of disability: arm amputee/leg amputee/paraplegic) between-and-within design that used photos and text descriptions of different people with disabilities as stimulus material as we explain below. We randomly assigned each participant to one of the three technicality between-participant conditions. In each condition, participants rated three individuals with the three different types of disabilities on warmth and competence. We included these different types of disabilities as a within-participant control condition to make sure that the results can be generalized beyond a certain specific type of physical disability.

#### 6.1.1. Participants

We conducted an a-priori power analysis with G^*^Power (Faul et al., 2006) for a within-and-between design with three measures for each participant and three experimental groups. We assumed a medium effect size of *f =* 0.25, an average correlation of *r =* 0.50 among measurements, and set the power to at least 0.95. This resulted in a required minimum sample size of 75 participants. We recruited these through Prolific Academic in the same way as described in Study 1. Because of the shorter questionnaire in Study 2 (average response time was 6 min), participants received 0.60 GBP for participation. We excluded individuals who had already participated in Study 1 from accessing Study 2. The ethics committee of our university reviewed the study before we conducted it and participants gave written informed consent.

As we had some remaining funds when conducting Study 2, we kept recruiting on Prolific Academic until these were fully used, resulting in 87 valid responses after screening out 22 participants who failed the IMC. These included 55 women and 30 men; 2 participants did not disclose their gender or selected another category. Average age was 33.45 years, *SD* = 11.88, and 10 participants reported that they had some form of recognized disability. The most frequent level of education was a Bachelor's degree, *n* = 21, followed by some college credit with no degree, *n* = 18, and high school graduate, diploma, or equivalent, *n* = 11. Regarding employment, 51 participants were employed for wages, *n* = 5 were self-employed, *n* = 14 were students, and *n* = 6 were homemakers. We randomly assigned participants to the three experimental conditions, which resulted in 26 participants in the little technicality, 30 in the medium technicality, and 31 in the high technicality condition.

#### 6.1.2. Material and procedure

We used photographs from the Alamy stock photography repository to manipulate the technical appearance of prostheses. A search of the repository with the terms “prostheses”, “amputee”, “disability”, “exo-skeleton”, and “paraplegic” resulted in a set of potential images. We chose nine images, each depicting a man in his 30–40s. One photo showed a man with a simple arm prosthesis, one showed a man with a more sophisticated arm prosthesis, and one showed a man with a high-tech bionic prosthesis^1^. We identified three similar pictures for leg prostheses. We also identified a photo of a man in a wheelchair, of the same man standing in a therapeutic exo-skeleton, and of another man standing in an advanced exo-skeleton. We bought the rights to use these photographs online and they are available on the OSF repository. To make the level of technicality of the depicted devices explicit, we presented a text vignette next to each photograph.

In the low technicality condition, the vignette read: “The person on the following image has a physical disability: [He is quadriplegic and uses a wheelchair/He is missing an arm and wears a body-powered prosthesis/He is missing a leg and wears a standard leg prosthesis] that restores little functionality in comparison with average able-bodied individuals.” In the medium technicality condition, the vignette read: “The person on the following image has a physical disability: [He is quadriplegic and uses an exoskeleton/He is missing an arm and wears a bionic hand prosthesis/He is missing a leg and wears a bionic leg prosthesis] that restores a fair amount of functionality in comparison with average able-bodied individuals.” In the high technicality condition, the text read “The person on the following image has a physical disability: [He is quadriplegic and uses an exoskeleton/He is missing an arm and wears a bionic prosthesis/He is missing both lower legs and wears bionic leg prostheses] that restore[s] functionality beyond the functions that average able-bodied individuals posses.”

Participants saw three pictures of individuals with different types of disability (paraplegic/arm amputee/leg amputee) with the same level of technicality in random order. After each image, we asked participants to rate the competence and warmth of the particular individual (“How do you perceive this person with regard to the following features?”), followed by the same six items as in Study 1. Omega Total reached 0.89 for warmth and 0.78 for competence, justifying averaging into the respective scales.

As in Study 1, to screen out random clicking and to ensure a thorough reading and understanding of each item, we used an instructional manipulation check. The item read “To screen out random clicking, please answer”not at all “to the feature “trustworthy”,” which we additionally presented underneath one of the photographs. Again, we warned participants about the IMC at the beginning of the questionnaire. The survey concluded with a page asking for participants' demographic data. All study materials are available from the OSF repository at https://osf.io/u2srb.

#### 6.1.3. Data analysis

We obtained three measurements of warmth and competence (for each of the three different types of disability) from each participant, totaling 250 measurements. Measurements are thus nested in participants and, consequently, likely to exhibit non-independence. Indeed, there was significant between-participant variability for warmth, ICC(1) = 0.74, *F*_(83, 160)_ = 9.479, *p* < 0.001, ICC(2) = 0.89, and for competence, ICC(1) = 0.37, *F*_(84, 165)_ = 2.691, *p* < 0.001, ICC(2) = 0.63. In other words, participants differed significantly with regard to the levels of warmth and competence that they generally attributed, which is why we employ mixed models as in Study 1.

### 6.2. Results

We fitted two mixed models with random intercepts regressing the two dependent variables, warmth and competence, on experimental condition, controlling for type of disability and participants' gender, age, and disability status. None of the control variables exhibited a significant effect on the dependent variables. We estimated both models' marginal means for the three experimental conditions, which we compared according to Hypotheses 5 and 6.

**Figure 2:**
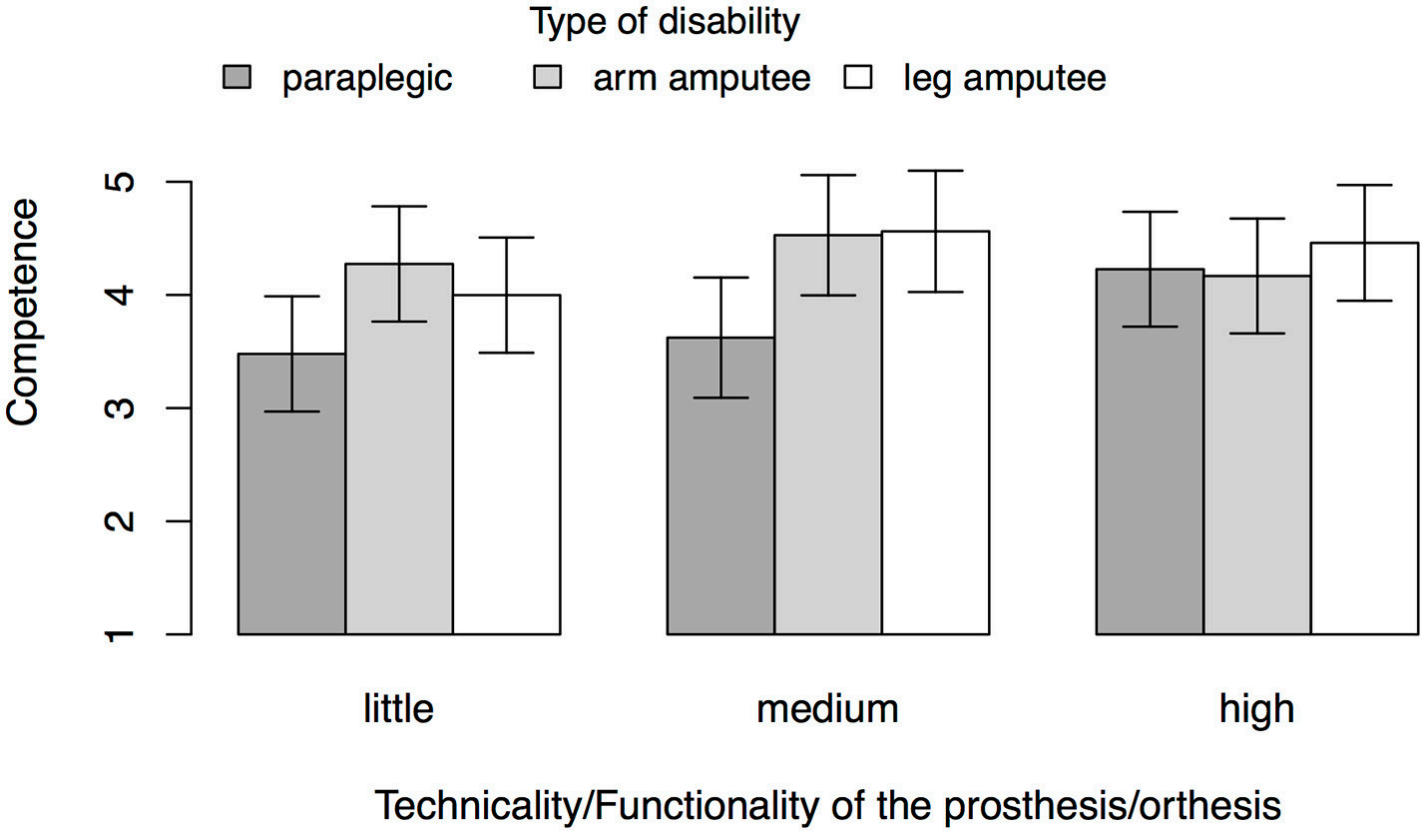
Estimated marginal means of competence for Study 2 controlling for participants' age, gender, and disability status. Values are based on 250 observations from 87 participants. Error bars represent 95% confidence intervals.

Specifically, Hypothesis 5 predicted that competence increases as a function of technicality of the prosthesis. Participants attributed an estimated marginal mean level of competence of *M* = 3.92 to targets in the little technicality condition, *SE* = 0.24, of *M* = 4.23 to targets in the medium technicality condition, *SE* = 0.26, and of *M* = 4.29 to targets in the high technicality condition, *SE* = 0.24. Three a-priori comparisons of these means with Shaffer control for alpha inflation revealed marginally significant differences between little and medium levels of technicality, Δ*M* = 0.31, σ = 0.16, *t* = 1.96, *p* = 0.074, and between little and high levels of technicality, Δ*M* = 0.37, σ = 0.16, *t* = 2.25, *p* = 0.074. The difference between high and medium levels of technicality was not significant, Δ*M* = −0.06, *p* = 0.71. The plot of the estimated marginal means of competence of all cells of the design revealed that these differences were primarily driven by perceptions of targets who are paraplegic and the targets with arm amputations (see Figure 2). In sum, these findings reveal a trend in the data that is partially in line with Hypothesis 5, but does not support it in the strict sense.

**Figure 3:**
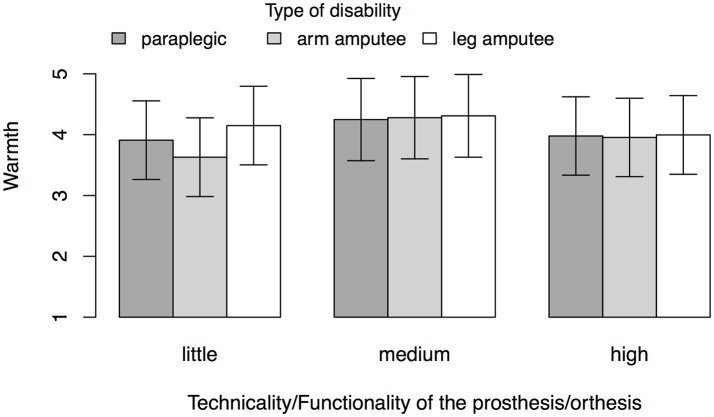
Estimated marginal means of warmth for Study 2 controlling for participants' age, gender, and disability status. Values are based on 250 observations from 87 participants. Error bars represent 95% confidence intervals.

Hypothesis 6 predicted that warmth decreases as a function of technicality of the prosthesis. Participants attributed an estimated marginal mean level of warmth of *M*= 3.90 to targets in the little technicality condition, *SE* = 0.32, of *M* = 4.28 to targets in the medium technicality condition, *SE* = 0.34, and of *M* = 3.98 to targets in the high technicality condition, *SE* = 0.32. Three a-priori comparisons of these means with Shaffer control for alpha inflation did not show significant differences, all *p*s ≥ 0.20. The absence of differences among conditions also becomes evident when plotting the estimated marginal means of warmth across all cells of the design (see Figure 3). We thus had to reject Hypothesis 6.

### 6.3. Discussion of study 2

We proposed that the different levels of perceivable technological sophistication of prosthetic and assistive devices affect attributions of warmth and competence toward those individuals who use them. To test this assumption, we tried to manipulate perceptions of technological sophistication by presenting images of different prostheses and exo-skeletons with different levels of technical advancements. However, in rejection of the hypotheses, the different levels of bionic sophistication visible in these images had no effect on participants' attributions of warmth, and only marginal effects on participants' attributions of competence. We can think of two possible explanations for these insignificant findings: First, other factors despite the technological appeal of the bionic devices affect attributions of warmth and competence. These may include the perceived abilities or activities of the disabled individual (cf. Barg et al., 2010) and further studies could test this possibility. Second, it may be possible that the manipulation was not strong enough and that the images did not convey different levels of technicality in an appropriate way. While we did try to steer perceptions of different levels of technicality with the text vignettes, these may have been insufficient. Future studies should therefore pre-test such figure materials. It is also possible that the overall posture or appearance of the individuals on the images had a strong effect on the attributions. Future research could address this issue by further systematizing the measurement of technology's appeal in terms of warmth and competence.

## 7. General discussion

We proposed that technological advancements in the area of bionics can affect the content of the stereotype toward people with physical disabilities. We hypothesized that people with physical disabilities who use bionic prostheses are perceived as more competent than people with physical disabilities in general, and that the same is true for “cyborgs”, a label that is sometimes used for this category. We also hypothesized that potential gains in attributed competence that people with physical disabilities who use bionic prostheses with regard to competence may be offset by potential losses on the warmth dimension. We also proposed that the technological appearance of the bionic devices affect attributions of competence and warmth toward the people who use them as a possible process behind the mechanisms that we propose.

The results from two studies offer mixed support for the hypotheses. The participants perceived people with physical disabilities who use bionic prostheses and cyborgs as much more competent than people with physical disabilities in general. However, participants did not perceive cyborgs and users of bionic prostheses as more competent than able-bodied individuals.

Higher levels of competence were associated with lower levels of attributed warmth for cyborgs. For people with physical disabilities who use bionic prostheses, this was not the case: Contrary to our hypotheses, they were even perceived as slightly warmer than able-bodied individuals (and almost as competent as them). In other words, people with physical disabilities who use bionic prostheses got to “keep” the warmth that is stereotypically associated with physical disabilities, but receive more competence through technology. This is insofar surprising as bionic prostheses are typically very expensive and are not covered by health insurances in some countries, which is why they could be seen as signaling wealth, which is typically construed as cold. This does however not seem to be the case. This finding shows that bionics can provide not only a functional benefit for people with disabilities, but also a psychological one: A bionic prosthesis can (partly) compensate for the lower levels of attributed competence that people with physical disabilities generally experience.

In line with labeling theory (Sapir, 1921) and previous findings showing that labels affect stereotype content (Kotzur et al., 2017), our study shows that attributions of warmth and competence depend on linguistic labels. People who use a bionic prosthesis for compensating limb loss can be called people with physical disabilities, people with physical disabilities who use bionic prostheses, and cyborgs. These labels carry different contents in terms of warmth and competence, as our study shows: Calling users of prostheses “cyborgs” makes them appear as competent and cold and therefore threatening, making envy and harm toward them more likely (Cuddy et al., 2007). This could even counter efforts aimed at creating more inclusive societies for all.

Apart from the rich and cyborgs, the groups that lie in the cold-and-competent area of Figure 1 also include able-bodied people who enhance themselves with technology. Interestingly, although the label implies that an enhancement beyond the abilities of able-bodied people took place, this group is not perceived as more competent than the able-bodied group. We can only speculate about the reason for this unexpected finding. As the results show, able-bodied people who enhance themselves are perceived as much colder than able-bodied people in general. Apparently, the attempt of the able-bodied to increase themselves is reprimanded by attributions of bad intentions. This can be seen as a punishment for violating a potential prescriptive norm associated with being able-bodied, namely to be happy with one's already privileged position in society. Attempts to increase this privilege even further through enhancement may result in a backlash, “social and economical reprisals for violating expectations that stereotypes carry” (van Dijk et al., 2017, p. 539). Potentially, this backlash is so strong that it spills into the competence dimension such that attempts to enhance oneself are perceived as not very competent. It is also possible that able-bodied people who enhance themselves are perceived as more threatening. According to previous SCM research (Kervyn et al., 2015), threat is negatively related to warmth perceptions and (less strongly) to competence perceptions. Regardless of the underlying mechanism, the findings pertaining to enhancement indicate that there is a social cost that goes along with potential enhancement.

### 7.1. Limitations

Our study is not without limitations. While the results indicate that technological advancements can affect stereotype content, our study stays mute to a number of underlying psychological processes. First, as we already discussed in the context of the non-significant findings of Study 2, the perceived technicality of the technology is not the core process behind the observed phenomena. Furthermore, in hindsight, one has to contend whether using stock photos showing the devices on different people in different contexts represented a good manipulation of technicality. Further research on the subject should consider other potential manipulations of the level of technicality of bionic devices.

Furthermore, we employed relatively broad categories in this research: The group “people with physical disabilities” is very wide and can include many different types of disabilities with varying degrees of impairments. Bionic technologies offer various degrees of assistance; a missing leg is easier to replace than eyesight, and the associated devices look very different. Future work could profit from employing a narrower focus on more specific disabilities and devices. Furthermore, when asking participants about their perception of people who use prostheses, we did not indicate whether the amputation was acquired or congenital. This distinction also might affect stereotypic attributions of warmth and competence; further studies on the matter could take this distinction into account. Future studies could also contain the information whether the high cost of a bionic prosthesis was covered by a health insurance or not. Future studies could also control for whether participants know someone who uses bionic prostheses or whether they believe in the abilities of this technology. We also have to contend that all our findings are about explicit stereotypes surrounding disability, bionics, and enhancement. Given that implicit stereotypes about people with disabilities tend to be more negative than explicit ones (Rohmer and Louvet, 2012), future studies need to show that bionics also affect implicit stereotypes.

Finally, our research stays mute with regard to the effects that media discourses surrounding bionics and enhancement play in the role of changing stereotypes. While we do assume that recent media discourses on these topics affect stereotype contents, we can offer only anecdotal evidence for these effects. Further studies, for example in the area of communication studies, could investigate the relationship between media coverage of, for example, the Paralympic games, and perceptions of people with disabilities to deeper extents (see Möller et al., 2011 for an analysis of implicit stereotypes of athletes with physical disabilities).

### 7.2. Conclusion and outlook

While the overall findings are somewhat mixed, to our knowledge, this is the first study showing that technology can affect stereotypes in society. We believe that this finding can add to a new line of research at the intersection of technology and (social) psychology that investigates how technological developments affect psychological processes (e.g., Bergmann et al., 2012). Further research could, for example, investigate attributions of warmth and competence to robots and computer programs to derive predictions of user reactions to the technology. Further research could also investigate the effect of other wearable technology, for example augmented reality glasses, on the perception of their wearers. In this way, psychology could contribute to our understanding of how technology affects interpersonal perceptions and interactions in the age of the digital transformation.

## Ethics statement

This study was carried out in accordance with the recommendations of the ethics committee of the faculty of behavioral and social sciences at Chemnitz University of Technology, Germany, and with the ethical guidelines of the American Psychological Association (APA) with written informed consent from all subjects. All subjects gave written informed consent in accordance with the Declaration of Helsinki. The protocol was reviewed by the ethics committee of the faculty of behavioral and social sciences at Chemnitz University of Technology, Germany.

The statement of the ethics committee - which decided that a full review is not necessary because the study was anonymous, voluntary, obtained written and informed consent, contained the possibility to withdraw later, did not recruit a vulnerable population, and did not involve any deceit or concealment of information. The committee's statement is available from the OSF repository at https://osf.io/9ejmd/ (Folder Pre-Registration, File Ergebnis_Vorbegutachtung.pdf).

## Author contributions

BM had the original idea for the study, collected and analyzed the data, and wrote the majority of the manuscript. FA helped with developing the specific hypotheses and study design and contributed to writing the introduction, theory section, and discussion.

### Conflict of interest statement

The authors declare that the research was conducted in the absence of any commercial or financial relationships that could be construed as a potential conflict of interest.

## Footnotes

^1^We were unable to find an image of a man wearing a high-tech arm prosthesis on Alamy. We thus conducted a wider internet image search and located a suitable image, for which we obtained permission from the photographer.
